# Association of the ADORA2A receptor and CD73 polymorphisms with epilepsy

**DOI:** 10.3389/fphar.2023.1152667

**Published:** 2023-03-29

**Authors:** Nan-Rui Shi, Qi Wang, Jie Liu, Ji-Zhou Zhang, Bin-Lu Deng, Xiu-Min Hu, Jie Yang, Xin Wang, Xiang Chen, Yan-Qin Zuo, Ting-Ting Liu, Jia-Ling Zheng, Xin Yang, Peter Illes, Yong Tang

**Affiliations:** ^1^ International Joint Research Centre on Purinergic Signalling, School of Acupuncture and Tuina/ School of Health and Rehabilitation, Chengdu University of Traditional Medicine, Chengdu, China; ^2^ Department of Neurology, Sichuan Provincial People’s Hospital, University of Electronic Science and Technology of China, Chengdu, China; ^3^ School of Clinical Medicine, Chengdu University of Traditional Medicine, Chengdu, China; ^4^ Rudolf Boehm Institute for Pharmacology and Toxicology, University of Leipzig, Leipzig, Germany; ^5^ Acupuncture and Chronobiology Key Laboratory of Sichuan Province, Chengdu, China

**Keywords:** A2A receptor, CD73, single-nucleotide polymorphism, epilepsy, purinergic receptor

## Abstract

Single-nucleotide polymorphisms are connected with the risk of epilepsy on occurrence, progress, and the individual response to drugs. Progress in genomic technology is exposing the complex genetic architecture of epilepsy. Compelling evidence has demonstrated that purines and adenosine are key mediators in the epileptic process. Our previous study found the interconnection of P2Y12 receptor single-nucleotide polymorphisms and epilepsy. However, little is known about the interaction between the purine nucleoside A_2A_ receptor and rate-limiting enzyme ecto-5′-nucleotidase/CD73 and epilepsy from the genetic polymorphism aspect. The aim of the study is to evaluate the impact of A_2A_R and CD73 polymorphisms on epilepsy cases. The study group encompassed 181 patients with epilepsy and 55 healthy volunteers. A significant correlation was confirmed between CD73 rs4431401 and epilepsy (*p* < 0.001), with TT genotype frequency being higher and C allele being lower among epilepsy patients in comparison with healthy individuals, indicating that the presence of the TT genotype is related to an increased risk of epilepsy (OR = 2.742, *p* = 0.006) while carriers of the C allele demonstrated a decreased risk of epilepsy (OR = 0.304, *p* < 0.001). According to analysis based on gender, the allele and genotype of rs4431401 in CD73 were associated with both male and female cases (*p* < 0.0001, *p* = 0.026, respectively). Of note, we found that A2AR genetic variants rs2267076 T>C (*p* = 0.031), rs2298383 C>T (*p* = 0.045), rs4822492 T>G (*p* = 0.034), and rs4822489 T>G (*p* = 0.029) were only associated with epilepsy in female subjects instead of male. It is evident that the TT genotype and T allele of rs4431401 in CD73 were genetic risk factors for epilepsy, whereas rs2267076, rs2298383, rs4822492, and rs4822489 polymorphisms of the A_2A_R were mainly associated with female subjects.

## Introduction

Single-nucleotide polymorphisms (SNPs) are valuable for diagnosis and treatment guidance in epilepsy (Pal et al., 2010). As one of the prominent forms of gene variations in the human genome (Kim and Misra, 2007), SNPs are utilized to detect encoded proteins for prevention and treatment in epilepsy genetic studies. With progress in genomic technology, almost a thousand genes have been verified to relate to epilepsy etiology (Wang et al., 2017). Observational publications have reported that the GABA receptor and GABA transporter-1 SNPs are associated with the risk of epilepsy (Sesarini et al., 2015; Schijns et al., 2020). Several autophagy-related protein 5 gene polymorphisms show significant associations with the susceptibility to late-onset epilepsy and temporal lobe epilepsy (Zhang et al., 2021). Additionally, there is an increasing focus on the role of purinergic signaling receptors in various central nervous system diseases, including epilepsy (Scheffer et al., 2017; Nikolic et al., 2020; Beamer et al., 2021). We reported in our former study that the polymorphisms of the P2Y12 receptor are related to epilepsy susceptibility, and one of the polymorphisms may be specifically associated with seizure frequency (Wang et al., 2022). CD73 plays a key role in ATP metabolism, which generates the adenosine that activates the A2AR. The overfunction of A2AR is sufficient to trigger brain dysfunction and induce neuronal excitotoxicity (Cunha et al, 2016). In addition, genetic deletion of CD73 was found to attenuate neuron degeneration in mice (Augusto et al., 2021). However, the relationship between epilepsy and SNPs from the purinergic signaling facet, particularly adenosine A_2A_ receptors (A_2A_R) and 5′-nucleotidase (CD73), currently has only a limited number of investigations.

It has already been widely studied that A_2A_R and CD73 participate in the etiology of epilepsy, whether in experimental or observational studies (Xu et al., 2022; El Yacoubi et al., 2009; Augusto et al., 2021). A_2A_R exists in both synapses and neurons (Rebola et al., 2005; Borea et al., 2018), is associated with adenylyl cyclase activation, and is thought to have an excitatory effect on neurons upon activation (Corvol et al., 2001). In the hippocampus of both animal models and those of human brains, A_2A_R upregulation in synapses has been demonstrated to be one of the pathogenic characteristics of epilepsy (Canas et al., 2018; Crespo et al., 2018; Barros-Barbosa et al., 2016). Patients with mesial temporal lobe epilepsy have an elevated proportion of A_2A_R during epileptogenesis, and this enhanced astrocytic A_2A_R has more public involvement in disorders linked to neuroexcitotoxicity (Barros-Barbosa et al., 2016). Neuronal excitation in epilepsy may increase synaptic A_2A_ activation, which aggravates synaptotoxicity and causes standard circuitry to deteriorate, resulting in epilepsy progression (Barros-Barbosa et al., 2016). In other words, A_2A_R overfunction plays an essential role in the cumulative aggravation of epilepsy rather than in the onset of seizure activity (Moreira-de-Sá et al., 2021). These studies support that A_2A_R may be involved in the pathophysiology of epilepsy by controlling the function of glial cells. Accordingly, the corresponding gene (ADORA2A) is studied as a promising candidate for epilepsy. ADORA2A is located on chromosome 22q 11.23 and has two coding exons spanning about 9 kb (MacCollin et al., 1994; Peterfreund et al., 1996). rs2298383 was proven to be associated with childhood epilepsy and a predisposition to childhood epilepsy (Fan et al., 2020). However, whether the association exists in a wider age range remains unclear. CD73 is an enzyme that catalyzes the last step in the extracellular metabolism of ATP to form adenosine (Alcedo et al., 2021) and is positioned ideally to promote A_2A_R activation after the conversion of released adenine nucleotides into adenosine (Cunha et al., 1996). A rodent study demonstrates that CD73 lost its activity along with the decreasing density of A_2A_R 48 h after hyperthermia-evoked convulsions. The amount and distribution of CD73 in the hippocampus of mesial temporal lobe epilepsy patients were higher and broader than that in control individuals, and hippocampal astrogliosis was observed in patients (Barros-Barbosa et al., 2016). Together, CD73 may be associated with epilepsy by promoting the A_2A_R activation after the conversion of released adenine nucleotides into adenosine. Furthermore, genetic variations in enzymes influencing extracellular adenosine homeostasis, including CD73, have been significantly associated with epilepsy. SNPs of CD73 have been significantly connected with epileptogenesis in the Caucasian race since variants may alter the function of CD73 to regulate the extracellular adenosine and seizure activity (Diamond et al., 2015), whereas the functions of ATP-related CD73 SNPs have not been completely illuminated in epileptic disease in the Asian race yet.

Given the substantiation that the adenosine A_2A_R and CD73 take part in the etiology of epilepsy in both clinical and rodent experiments, we designed this study to investigate changes between epilepsy cases and control individuals and from genetic variations aspects which are worth exploring as therapeutic targets for treatment development.

## Participants and methods

### Subjects

Between August 2020 and August 2021, 181 epilepsy cases (92 male and 89 female) were diagnosed according to the 2014 International League against Epilepsy criteria (Thijs et al., 2019), and there were 50 healthy participants (22 male and 28 female). The medians (ranges of the first quartile to the third quartile) of age for the cases and volunteers were 28 (23–47) and 26 (25–28), respectively. The subjects were recruited at the Sichuan Academy of Medical Science and Sichuan Provincial People’s Hospital in China. Clinical data of patients were collected, including gender, age, disease diagnosis, seizure onset frequency, medical history, drug treatment, and imaging examination. Individuals with missing abovementioned clinical data were excluded from the study. Those with a history of pseudo-epileptic seizures, as well as with impaired hepatic and/or renal function, were excluded. Healthy controls were neurologically normal, with no personal or family history of epilepsy.

Following approval of the Sichuan Academy of Medical Science and Sichuan Provincial People’s Hospital Ethics Committees, written informed consent was obtained from the individuals before participation in the study. Blood samples were taken with the consent of the individuals, and 2 ml blood from each participant was collected in EDTA tubes and kept at −20°C for extraction of DNA and genotyping. Samples were stored at −70°C until analysis.

### DNA extraction and genotyping

Genomic DNA was isolated from peripheral blood using a QIAGEN kit (QIAGEN, Hilden, Germany). Extracted DNA was quantified using a NanoDrop analyzer (ND-2000) spectrophotometer (NanoDrop Technologies Inc., Wilmington, DE, United States). Qualified DNA samples were stored at −80°C until further use.

We selected fourteen single-nucleotide polymorphisms from two genes involved in the adenosine cycle for analysis. Genotyping of CD73 rs4431401 T>C, rs2065114 A>G, rs2229523 A>G, rs4579322 T>A, rs9444348 G>A, rs9450282 A>G, rs6922 T>G, rs4373337 A>C, rs2267076 T>C, and A_2A_R rs3761422 T> rs2298383 C>T, rs4822492 C>G, rs2236624 T>C, and rs4822489 T>G polymorphisms was performed by the MassARRAY platform (Agena Bioscience, San Diego, CA, United States) at CapitalBio (Beijing, China). The primers for PCR amplification and extension were designed using the MassARRAY Assay Design v4.0 software. The PCR cycle program, as well as shrimp alkaline phosphatase digestion and extension, was performed according to the manufacturer’s protocol. Extension products were desalted and detected using matrix-assisted laser desorption ionization time-of-fight. Finally, the data were processed with Typer v4.0 software (Agena Bioscience, San Diego, CA, United States).

### Statistical analysis

Statistical analysis was performed using the Statistical Package for the Social Sciences (SPSS), version 26.0 (IBM, Chicago, IL, United States). Categorical variables were expressed as numbers and percentages and compared by using the Pearson chi-squared test and Fisher’s exact test. Numerical variables were expressed as medians with interquartile ranges and compared by the non-parametric independent-sample Wilcoxon signed-rank test. The χ^2^ test was used to assess the deviation from the Hardy–Weinberg equilibrium. The χ^2^ statistics or Fisher’s exact test was used to compare the statistical differences in genotype distributions and allele frequencies between the cases and controls. The odds ratio (OR) was calculated with 95% confidence intervals (CIs). Statistical significance was defined as two-tailed *p* < 0.05.

## Results

Genotype frequencies of all investigated ADORA2A and CD73 SNPs conformed to the Hardy–Weinberg equilibrium in epilepsy and the healthy control samples.

### Clinical characteristics of the study participants

Demographic and clinical characteristics of the 231 enrolled participants are shown in Table 1.

**TABLE 1:** Demographic and clinical characteristics of the enrolled population.

Variables	Epileptic patients (n=181)	Healthy controls (n=50)	*p* value
Age(years)	28 (23-47)	26 (25-28)	0.079
Gender			
Male	92 (50.5%)	22 (44.0%)	0.427
Female	89 (49.5%)	28 (56.0%)	—
Drug treatment			
Monotherapy	122 (67.4%)	—	—
Polytherapy	57 (31.5%)	—	—
No	2 (1.1%)	—	—
Neuroimaging			
Abnormal	80 (44.2%)	—	—
Normal	101 (55.8%)	—	—
Epileptic seizure frequencies			
< 2 times/year	80 (44.2%)	—	—
≥ 2 times/year	101 (55.8%)	—	—

The study included 181 patients (92 male and 89 female) and 50 volunteers (22 male and 28 female) with median ages of 28 years and 26 years, respectively. There were no statistically significant differences between epileptic patients and healthy controls in terms of gender or age.

### Genotypic and allelic distribution of the CD73 and A_2A_R SNPs

The frequency distributions of the CD73 and A_2A_R polymorphisms were compared between epilepsy patients and healthy controls. Of the fourteen investigated SNPs, a significant difference was observed in the CD73 SNP rs4431401. The genotype frequencies of CD73 rs4431401 T>C polymorphism CC, CT, and TT genotypes were found in 12.7%, 40.9%, and 46.4% of cases and 48%, 28%, and 24% of the control group, respectively. The allelic frequency was 33.1% for the C allele and 66.9% for the T allele in patients, while it was 62% for the C allele and 38% for the T allele in the volunteer group. The T allele and TT genotype were conspicuously higher among patients than in healthy controls (OR = 0.305, 95% CI = 0.193–0.483, *p* = 0.0001 for C vs. T; OR = 2.714, 95% CI = 1.333–5.529, *p* = 0.006 for TT vs. CT/CC), indicating that individuals with the T allele and TT genotype of rs4431401 T>C might have higher risks for epilepsy (Table 2). However, the risk of epilepsy did not differ in other CD73 and A_2A_R polymorphisms between the cases and the control group (Supplementary Tables S1, S2).

**TABLE 2:** Genotypic and allelic distribution of the CD73 gene between all patients and controls.

SNP ID	Genetic model	Genotype/allele	Cases	Controls	OR	95% CI	*p value*
rs4431401	Codominant	CC vs. CT vs. TT	23 (12.7%)/74 (40.9%)/84 (46.4%)	24 (48%)/14 (28%)/12 (24%)	–	–	0.000*
	Allele contrast	C vs. T	120 (33.1%)/242 (66.9%)	62(62%)/38(38%)	0.304	0.192–0.481	0.000*
	Dominant	TT vs. CT+CC	84 (46.4%)/97 (53.6%)	12 (24%)/38 (76%)	2.742	1.346–5.587	0.006*
	Recessive	CT+TT vs. CC	158 (87.3%)/23 (12.7%)	26(52%)/24(48%)	6.341	3.129–12.853	0.000*
	Overdominant	CC+TT vs. CT	107 (59.1%)/74 (40.9%)	36 (72%)/14 (28%)	0.562	0.284–1.115	0.103

### Genotypic and allelic distribution of CD73 and A_2A_R SNPs in different genders

The CD73 and A_2A_R genotype and allele frequencies between the patients and controls of different genders are summarized in Table 3, Table 4, and Supplementary Tables S3 and S4. We found that the frequencies of the alleles and genotypes in CD73 rs4431401 between both male and female groups varied significantly (*p* < 0.001). With the T allele and TT genotype frequency being lower among the healthy in comparison with the epilepsy subjects, the presence of the T allele and TT genotype was connected with an increased risk of epilepsy (OR = 0.149, CI = 0.069–0.322, *p* < 0.001; OR = 5.092, CI = 1.408–18.407, *p* = 0.013, respectively) (Table 3). Females carrying the C allele/CC variant in CD73 rs4431401 had a lower risk of epilepsy in contrast with females who carried no copies (OR = 0.483, 95% CI = 0.263–0.890, *p* = 0.026 for C vs. T; OR = 3.039, 95% CI = 1.119–8.258, *p* = 0.045 for CT/TT vs. CC) (Table 3). No additional significant genotypic and allelic distribution between the female and male patients was observed in our study (Supplementary Table S3).

**TABLE 3:** Genotypic and allelic distribution of the CD73 gene between all patients and controls in different genders.

SNP ID	Gender	Genetic model	Genotype/allele	Cases	Controls	OR	95% CI	*p* value
rs4431401	Male	Codominant	CC vs. CT vs. TT	11(12%)/40(43.5%)/41(44.6%)	15(68.2%)/4(18.2%)/3(13.6%)	–	–	0.000*
		Allele contrast	C vs. T	62(33.7%)/122(66.3%)	34(77.3%)/10(22.7%)	0.149	0.069–0.322	0.000*
		Dominant	TT vs. CT+CC	41(44.6%)/51(55.4%)	3(13.6%)/19(86.4%)	5.092	1.408–18.407	0.013*
		Recessive	CT+TT vs. CC	81(88%)/11(12%)	7(31.8%)/15(68.2%)	15.779	5.273–47.221	0.000*
		Overdominant	CC+TT vs. CT	52(56.5%)/40(43.5%)	18(81.8%)/4(18.2%)	0.289	0.091–0.921	0.049*
	Female	Codominant	CC vs. CT vs. TT	12(13.5%)/34(38.2%)/43(48.3%)	9(32.1%)/10(35.7%)/9(32.1%)	–	–	0.067
		Allele contrast	C vs. T	58(32.6%)/120(67.4%)	28(50%)/28(50%)	0.483	0.263–0.890	0.026*
		Dominant	TT vs. CT+CC	43(48.3%)/46(51.7%)	9(32.1%)/19(67.9%)	1.973	0.806–4.832	0.190
		Recessive	CT+TT vs. CC	77(86.5%)/12(13.5%)	19(67.9%)/9(32.1%)	3.039	1.119–8.258	0.045*
		Overdominant	CC+TT vs. CT	55(61.8%)/34(38.2%)	18(64.3%)/10(35.7%)	0.899	0.371–2.174	0.828

**TABLE 4:** Genotypic and allelic distribution of the A2AR gene between all patients and controls in different genders.

SNP ID	Gender	Genetic model	Genotype/allele	Cases	Controls	OR	95% CI	*p value*
rs2267076	Female	Codominant	CC vs. TC vs. TT	32(35%)/49(55.1%)/8(9%)	14(50%)/8(28.6%)/6(21.4%)	–	–	0.031*
		Allele contrast	C vs. T	113(63.5%)/65(36.5%)	36(64.3%)/20(35.7%)	0.966	0.516-1.806	1
		Dominant	TT vs. TC+CC	8(9%)/81(91%)	6(21.4%)/22(78.6%)	0.362	0.114-1.154	0.097
		Recessive	TC+TT vs. CC	57(64%)/32(36%)	14(50%)/14(50%)	1.781	0.755-4.201	0.267
		Overdominant	CC+TT vs. TC	40(44.9%)/49(55.1%)	20(71.4%)/8(28.6%)	0.327	0.130-0.819	0.017*
	Male	Codominant	CC vs. TC vs. TT	37(40.2%)/37(40.2%)/18(19.6%)	7(31.8%)/10(45.5%)/5(22.7%)	–	–	0.822
		Allele contrast	C vs. T	111(60.3%)/73(39.7%)	24(54.5%)/20(45.5%)	1.267	0.653-2.459	0.499
		Dominant	TT vs. TC+CC	18 (19.6%)/74(80.4%)	5(22.7%)/17(77.3%)	0.827	0.289-2.541	0.770
		Recessive	TC+TT vs. CC	55(59.8%)/37(40.2%)	15(68.2%)/7(31.8%)	0.694	0.258-1.865	0.627
		Overdominant	CC+TT vs. TC	55 (59.8%)/37(40.2%)	12(54.5%)/10(45.5%)	1.239	0.485-3.162	0.810
rs2298383	Female	Codominant	CC vs. CT vs. TT	16(18%)/52(58.4%)/21(23.6%)	7(25%)/9(32.1%)/12(42.9%)	–	–	0.045*
		Allele contrast	C vs. T	84(47.2%)/94(52.8%)	23(41.1%)/33(58.9%)	1.282	0.689-2.356	0.446
		Dominant	TT vs. CT+CC	21(23.6%)/68(76.4%)	12(42.9%)/16(57.1%)	0.412	0.168-1.007	0.057
		Recessive	CT+TT vs. CC	73(82%)/16(18%)	21(75%)/7(25%)	1.521	0.553-4.185	0.587
		Overdominant	CC+TT vs. CT	37(41.6%)/52(58.4%)	19(67.9%)/9(32.1%)	0.337	0.137-0.827	0.018*
	Male	Codominant	CC vs. CT vs. TT	27(29.3%)/40(43.5%)/25(27.2%)	5(22.7%)/10(45.5%)/7(31.8%)	–	–	0.870
		Allele contrast	C vs. T	94(51.1%)/90(48.9%)	20(45.5%)/24(54.5%)	1.253	0.648-2.425	0.615
		Dominant	TT vs. CT+CC	25(27.2%)/67(72.8%)	7(31.8%)/15(68.2%)	0.800	0.292-2.191	0.792
		Recessive	CT+TT vs. CC	65(70.7%)/27(29.3%)	17(77.3%)/5(22.7%)	0.708	0.237-2.113	0.608
		Overdominant	CC+TT vs. CT	52(56.5%)/40(43.5%)	12(54.5%)/10(45.5%)	1.083	0.425-2.759	1
rs4822492	Female	Codominant	CC vs. CG vs. GG	16(17%)/53(60.2%)/20(22.7%)	7(25%)/9(32.1%)/12(42.9%)	–	–	0.034*
		Allele contrast	C vs. G	85 (47.8%)/93(52.2%)	23(41.1%)/33(58.9%)	1.311	0.714-2.409	0.443
		Dominant	GG vs. CG+CC	20(22.5%)/69(77.5%)	12(42.9%)/16(57.1%)	0.386	0.157-0.949	0.051
		Recessive	CG+GG vs. CC	73(82%)/16(18%)	21(75%)/7(25%)	1.521	0.553-4.185	0.587
		Overdominant	CC+GG vs. CG	36(40.4%)/53(59.6%)	19(67.9%)/9(32.1%)	0.322	0.131-0.791	0.016*
	Male	Codominant	CC vs. CG vs. GG	26(28.3%)/41(44.6%)/25(25.8%)	5(22.7%)/10(45.5%)/7(31.8%)	–	–	0.872
		Allele contrast	C vs. G	93(50.5%)/91(49.5%)	20(45.5%)/24(54.5%)	1.226	0.634-2.373	0.616
		Dominant	GG vs. CG+CC	25(27.2%)/67(72.8%)	7(31.8%)/15(68.2%)	0.800	0.292-2.191	0.792
		Recessive	CG+GG vs. CC	66(71.7%)/26(28.3%)	17(77.3%)/5(22.7%)	0.747	0.250-2.233	0.791
		Overdominant	CC+GG vs. CG	51(55.4%)/41(44.6%)	12(54.5%)/10(45.5%)	1.037	0.407-2.639	1
rs4822489	Female	Codominant	GG vs. GT vs. TT	20(22.7%)/53(60.2%)/16(17%)	12(27.6%)/9(53.4%)/7(19%)	–	–	0.034*
		Allele contrast	G vs. T	93(52.2%)/85(47.8%)	33(58.9%)/23(41.1%)	0.763	0.415-1.401	0.443
		Dominant	TT vs. GT+GG	16(18%)/73(82%)	7(25%)/21(75%)	0.658	0.239-1.809	0.587
		Recessive	GT+TT vs. GG	69(77.5%)/20(22.5%)	16(57.1%)/12(42.9%)	2.588	1.053-6.357	0.051
		Overdominant	GG+TT vs. GT	36(40.4%)/53(59.6%)	19(67.9%)/9(32.1%)	0.322	0.131-0.791	0.016*
	Male	Codominant	GG vs. GT vs. TT	25(27.2%)/41(44.6%)/26(28.3%)	7(31.8%)/10(45.5%)/5(22.7%)	–	–	0.872
		Allele contrast	G vs. T	91(49.5%)/93(50.5%)	24(54.5%)/20(45.5%)	0.815	0.421-1.578	0.616
		Dominant	TT vs. GT+GG	26(28.3%)/66(71.7%)	5(22.7%)/17(77.3%)	1.339	0.448-4.006	0.791
		Recessive	GT+TT vs. GG	67(72.8%)/25(27.2%)	15(68.2%)/7(31.8%)	1.251	0.456-3.427	0.792
		Overdominant	GG+TT vs. GT	51(55.4%)/41(44.6%)	12(54.5%)/10(45.5%)	1.037	0.407-2.639	1

In the A_2A_R gene, there were no associations of the identified SNPs, rs3761422 and rs2236624, with analysis based on gender (Supplementary Table S4). Interestingly, we observed that SNPs for the A_2A_R gene differed between female cases and controls, including rs2267076 T>C (*p* = 0.031), rs2298383 C>T (*p* = 0.045), rs4822492 T>G (*p* = 0.034), and rs4822489 T>G (*p* = 0.034). The differences were mainly attributed to a greater proportion of heterozygotes and fewer homozygotes. They were observed in A_2A_R gene polymorphisms, where female cases with CT in rs2267076 (OR = 0.327, CI = 0.130–0.819, *p* = 0.017), TC in rs2298383 (OR = 0.337, CI = 0.137–0.827, *p* = 0.018), CG in rs4822492 (OR = 0.322, CI = 0.131–0.791, *p* = 0.016), and GT in rs4822489 (OR = 0.322, CI = 0.131–0.791, *p* = 0.016) had a higher proportion of heterozygotes than homozygotes (Table 4). Therefore, females who are heterozygous genotype carriers of rs2267076, rs229838, rs4822489, and rs4822492 polymorphisms have a higher risk of epilepsy.

### Subgroup analysis of A_2A_R and CD73

We conducted sub-analyses to determine whether risk varied by subgroups differing in drug treatment, neuroimaging, or epileptic seizure frequencies. We did not find any significant connection between neuroimaging and seizure frequency subgroups but did find an association between genotypes and drug therapy among epilepsy cases (Supplementary Tables S5, S6). This relationship was evident only for single-nucleotide polymorphisms on the A_2A_R gene (Table 5). The frequency of the TT genotype (38.6% for polytherapy; 46.7% for monotherapy) and T allele (60.5% for polytherapy; 46.7% for monotherapy) for rs2298383 in the A_2A_R gene was higher among cases of polypharmacy than in single drug treatment groups, suggesting that patients that had the TT genotype and T allele (TT vs. CT/CC: OR = 0.390, CI = 0.194–0.781, *p* = 0.010; C vs. T: OR = 1.749, CI = 1.113–2.478, *p* = 0.017) had a higher potential of requiring two or more antiepileptic drugs. Similar to former results, in rs4822492, the GG genotype and G allele were associated with polytherapy (GG vs. CG/CC: OR = 0.370, CI = 0.184–0.744, *p* = 0.006; C vs. G: OR = 1.844, CI = 1.172–2.902, *p* = 0.009). In addition, we found that patients carrying the GG variant in A_2A_R rs4822489 were associated with polypharmacy (OR = 2.706, CI = 1.343–5.449, *p* = 0.006).

**TABLE 5:** Genotypic and allelic distribution of CD73 and A2AR genes between patients with monotherapy and polytherapy.

Gene	SNP ID	Genetic model	Genotype/allele	Monotherapy	Polytherapy	OR	95% CI	*p value*
CD73	rs4431401	Codominant	CC vs. CT vs. TT	14 (11.5%)/51 (41.8%)/57 (46.7%)	9 (15.8%)/22 (38.6%)/26 (45.6%)	–	–	0.733
		Allele contrast	C vs. T	79 (32.4%)/165 (67.6%)	40 (35.1%)/74 (64.9%)	0.886	0.554–1.416	0.631
		Dominant	TT vs. CT+CC	57 (46.7%)/65 (53.3%)	26 (45.6%)/31 (54.4%)	1.046	0.556–1.965	1
		Recessive	CT+TT vs. CC	108 (88.5%)/14 (11.5%)	48 (84.2%)/9 (15.8%)	1.446	0.586–3.571	0.474
		Overdominant	CC+TT vs. CT	71 (58.2%)/51 (41.8%)	35 (61.4%)/22 (38.6%)	0.875	0.460–1.665	0.745
A2AR	rs2267076	Codominant	CC vs. TC vs. TT	41 (33.6%)/63 (51.6%)/18 (14.8%)	27 (47.4%)/23 (40.4%)/7 (12.3%)	–	–	0.222
		Allele contrast	C vs. T	145 (59.4%)/99 (40.6%)	77 (67.5%)/37 (32.5%)	0.704	0.441–1.124	0.161
		Dominant	TT vs. TC+CC	18 (14.8%)/104 (85.2%)	7 (12.3%)/50 (87.7%)	1.236	0.485–3.152	0.818
		Recessive	TC+TT vs. CC	81 (66.4%)/41 (33.6%)	30 (52.6%)/27 (47.4%)	1.778	0.936–3.377	0.098
		Overdominant	CC+TT vs. TC	59 (48.4%)/63 (51.6%)	34 (59.6%)/23 (40.4%)	0.634	0.335–1.198	0.199
A2AR	rs2298383	Codominant	CC vs. CT vs. TT	32 (26.2%)/66 (54.1%)/24 (19.7%)	10 (17.5%)/25 (43.9%)/22 (38.6%)	–	–	0.023*
		Allele contrast	C vs. T	130 (53.3%)/114 (46.7%)	45 (39.5%)/69 (60.5%)	1.749	1.113–2.748	0.017*
		Dominant	TT vs. CT+CC	24 (19.7%)/98 (80.3%)	22(38.6%)/35(61.4%)	0.390	0.194–0.781	0.010*
		Recessive	CT+TT vs. CC	90 (73.8%)/32 (26.2%)	47 (82.5%)/10 (17.5%)	0.598	0.271–1.322	0.257
		Overdominant	CC+TT vs. CT	56 (45.9%)/66 (54.1%)	32 (56.1%)/25 (43.9%)	0.663	0.352–1.248	0.261
A2AR	rs4822492	Codominant	CC vs. CG vs. GG	32 (26.2%)/67 (54.9%)/23 (18.9%)	9 (15.8%)/26 (45.6%)/22 (38.6%)	–	–	0.013*
		Allele contrast	C vs. G	131 (53.7%)/113 (46.3%)	44 (38.6%)/70 (61.4%)	1.844	1.172–2.902	0.009*
		Dominant	GG vs. CG+CC	23 (18.9%)/99 (81.1%)	22 (38.6%)/35 (61.4%)	0.370	0.184–0.744	0.006*
		Recessive	CG+GG vs. CC	90 (73.8%)/32 (26.2%)	48 (84.2%)/9 (15.8%)	0.527	0.233–1.195	0.132
		Overdominant	CC+GG vs. CG	55 (45.1%)/67 (54.9%)	31 (54.4%)/26 (45.6%)	0.688	0.366–1.295	0.265
A2AR	rs4822489	Codominant	GG vs. GT vs. TT	23 (18.9%) 67 (54.9%)/32 (26.2%)	22 (38.6%)/26 (45.6%)/9 (15.8%)	–	–	0.013*
		Allele contrast	G vs. T	113 (46.3%)/131 (53.7%)	70(61.4%)/44(38.6%)	0.542	0.345-0.853	0.009
		Dominant	TT vs. GT+GG	32 (26.2%)/90 (73.8%)	9 (15.8%)/48 (84.2%)	1.896	0.837–4.298	0.132
		Recessive	GT + TT vs. GG	99 (81.1%)/23 (18.9%)	35 (61.4%)/22 (38.6%)	2.706	1.343–5.449	0.006*
		Overdominant	GG + TT vs. GT	55 (45.1%)/67 (54.9%)	31 (54.4%)/26 (45.6%)	0.688	0.366–1.295	0.265

## Discussion

We tested the hypothesis that adenosine-related SNPs are connected with the risk of epilepsy.

Our data showed that the T allele and TT genotype of SNP rs4431401 of CD73 was associated with a more pronounced predisposition to an increased risk of epilepsy, while A_2A_R gene rs2267076, rs2298383, rs4822492, and rs4822489 polymorphisms were more strongly linked with female epileptic patients. In contrast, there was no evidence for interactions of the identified SNPs: rs2065114, rs2229523, rs4579322, rs9444348, rs9450282, rs6922, and rs4373337 of CD73, rs3761422, and rs2236624 of A_2A_R. Noteworthily, rs2298383, rs4822492, and rs4822489 on the A_2A_R gene were associated with medication administered among epilepsy cases. These findings provide insight into the genetic susceptibility of epileptic disease and assistance for clinical drug therapy.

We found that carriers of rs4431401 in CD73, with a higher proportion of T allele and TT genotype, may have a higher predisposition for epilepsy. Observational studies focused on nephrotic syndrome (NS) (Yang et al., 2018; Zaorska et al., 2021) and uremia patients (Rothe et al., 2017) and found that rs4431401 (T>C) was significantly correlated with both differed NS risk and altered hormone sensitivity to NS. We also observed that both female and male subjects have a higher frequency of TT genotype compared to the controls. Since the risk factors of epilepsy are complex, such as family history, excessive sleep deprivation, and use of alcohol (Gavvala and Schuele, 2016), we speculate that one of the possible mechanisms is that gender difference influences the cognitive strategies on brain activation, such as women preferring the left hemisphere while men favoring the right hemisphere (Koepp, 2011). In addition, endogenous sex hormones may play a role. In an earlier cohort study, rs9444348 of CD73 was reported to have been significantly associated with a shorter time to first seizure and an increased seizure rate within 3 years of post-traumatic brain injury in Caucasian patients (Diamond et al., 2015). However, no difference was found in our present study. Race, the pathogenesis of epilepsy, and the statistical method were regarded to be the feasible reasons which are causing differences in results.

Females who carried a greater proportion of heterozygotes in rs2298383, rs2267076, rs4822492, and rs4822489 polymorphisms on the A_2A_R gene were identified to have an increased risk of epilepsy. On the contrary, no significant difference was observed in male patients. Consistent with this, a rodent study showed that female rats were more susceptible to acquiring seizures than male rats (Dai et al., 2014). Since sex hormones are associated with neuronal development, neuronal excitability, and epileptic susceptibility (Patrone et al., 1999; Zupanc, 2006; Liu et al., 2012), the possible reason is the effect of endogenous sex hormones, such as androgen, estrogen, and progesterone, as well as their metabolites. In addition, a recent study based on southern Chinese children with epileptic diseases shows that the carriers of the rs2298383 TT genotype tended to have a lower chance of epilepsy (Fan et al., 2020). In addition, the haplotype C frequency at rs2298383 and rs4822492 polymorphisms was reported to be significantly higher than in controls in acute encephalopathy with biphasic seizures and late reduced diffusion in children from Japan (Shinohara et al., 2013). The fact that these results are incompatible with ours could be attributed to the differences in age and the regions from where the participants were enrolled. As for s2267076 and rs4822489, we found no directly comparable studies when we searched PubMed for studies published in English that investigated the association between them and the risk of epilepsy. We have, therefore, provided comparisons with broader literature. The current study indicates that a higher risk of rheumatoid arthritis was found in patients who consume more caffeine with a CT genotype of rs2267076 (Soukup et al., 2020). rs4822489 is associated with chronic heart failure and type 1 diabetes (Charles et al., 2011; Zhai et al., 2015). To our knowledge, the genetic polymorphisms rs2267076 and rs4822489 were first identified to be associated with epilepsy in the present study.

We also observed an increased risk of polypharmacy in individuals with the TT genotype and T allele of rs2298383. An earlier case-control study reported that rs2298383 polymorphisms associated with tear volume increase after caffeine intake (Arita et al., 2012). Caffeine has various pharmacologic effects on the human body, including stimulation of the central nervous system (van Dam et al., 2020). A_2A_R, as one of the main target receptors of caffeine, has already been proven to play an important role in caffeine metabolism (Cappelletti et al., 2015). In addition, genetic factors are revealed to be associated with the direct effects of caffeine (Yang et al., 2010). In this way, an in-depth study needs to investigate whether rs2298383 is linked with polypharmacy of antiepileptic drugs and caffeine. In addition, though our results suggest that A_2A_R and CD73 gene polymorphisms do not correlate with the epileptic seizure frequency or abnormal/normal neuroimaging, they still require further investigation.

Some potential limitations should be considered. First, we only analyzed the population in southwestern China because representation from other regions of the country is lacking. Future studies should involve patients from the greater China region. Second, the SNPs of CD73 have been rarely reported in epilepsy. Hence, the discussion concerning the SNPs of CD73 is limited. Further validation and studies are necessary to confirm the relationship between CD73 and epilepsy. Finally, this study was confined to the association of SNPs with epilepsy and lacked specific epileptic sub-types due to the limited sample size. Therefore, more in-depth research is needed to improve our understanding of the association between CD73 and A_2A_ receptors and the pathophysiology of epilepsy.

## Data Availability

The original contributions presented in the study are included in the article/Supplementary Material; further inquiries can be directed to the corresponding authors.
