# Supplementary material for: Association of the ADORA2A receptor and CD73 polymorphisms with epilepsy
Source: Front Pharmacol. 2023 Mar 29;14:1152667. doi: 10.3389/fphar.2023.1152667 (PMC10090369; doi:10.3389/fphar.2023.1152667)
Supplement: Supplementary file 1 [file Presentation1.pdf]

Supplementary

Table 1 Genotypic and allelic distribution of the CD73 gene between all patients and controls

SNP ID	Genetic model	Genotype/allele	Cases	Controls	OR	95%CI	*p value*
rs2065114	Codominant	AAvs.GA vs.GG	29(16%)/89(49.2%)/63(34.8%)	7(14%)/24(48%)/19(38%)	-	-	0.929
	Allele contrast	A vs. G	147(40.6%)/215(59.4%)	38(38%)/62(62%)	1.116	0.708-1.758	0.647
	Dominant	GG vs. GA+AA	63(34.8%)/118(65.2%)	19(38%)/31(62%)	0.871	0.456-1.665	0.739
	Recessive	GA+GG vs. AA	152(84%)/29(16%)	43(86%)/7(14%)	0.853	0.350-2.082	0.828
	Overdominant	AA+GG vs. GA	92(50.8%)/89(49.2%)	26(52%)/24(48%)	0.954	0.510-1.786	1.000
rs2229523	Codominant	AAvs.AG vs.GG	28(15.5%)/88(48.6%)/65(35.9%)	6(12%)/21(42%)/23(46%)	-	-	0.431
	Allele contrast	A vs. G	144(39.8%)/218(60.2%)	33(33%)/67(67%)	1.341	0.841-2.139	0.246
	Dominant	GG vs. AG+AA	65(35.9%)/116(64.1%)	23(46%)/27(54%)	0.658	0.349-1.240	0.249
	Recessive	AG+GG vs. AA	153(84.5%)/28(15.5%)	44(88%)/6(12%)	0.745	0.290-1.914	0.655
	Overdominant	AA+GG vs. AG	93(51.4%)/88(48.6%)	29(58%)/21(42%)	0.765	0.406-1.441	0.428
rs4579322	Codominant	AA vs. AT vs.TT	63(34.8%)/89(49.2%)/29(16%)	19(38%)/24(48%)/7(14%)	-	-	0.929
	Allele contrast	A vs. T	215(59.4%)/147(40.6%)	62(62%)/38(38%)	0.896	0.569-1.413	0.647
	Dominant	TT vs. AT+AA	29(16%)/152(84%)	7(14%)/43(86%)	1.172	0.480-2.860	0.828
	Recessive	AT+TT vs. AA	118(65.2%)/63(34.8%)	31(62%)/19(38%)	1.148	0.601-2.194	0.739
	Overdominant	AA+TT vs. AT	92(50.8%)/89(49.2%)	26(52%)/24(48%)	0.954	0.510-1.786	1.000
rs9444348	Codominant	AAvs.AG vs.GG	23(12.7%)/70(38.7%)/88(48.6%)	5(10%)/23(46%)/22 (44%)	-	-	0.676
	Allele contrast	A vs. G	116(32%)/246(68%)	33(33%)/67 (67%)	0.957	0.597-1.534	0.904
	Dominant	GG vs. AA+AG	88(48.6%)/93(51.4%)	22(44%)/28(56%)	1.204	0.641-2.261	0.632

	Recessive	AG+GG vs. AA	158(87.3%)/23(12.7%)	45(90%)/5(10%)	0.763	0.275-2.122	0.640
	Overdominant	AA+GG vs.AG	111(61.3%)/70(38.7%)	27(54%)/23(46%)	1.351	0.718-2.540	0.416
rs9450282	Codominant	AA vs.AGvs.GG	79(43.6%)/74(40.9%)/28(15.5%)	18(36%)/26(52%)/6(12%)	-	-	0.383
	Allele contrast	A vs. G	232(64.1%)/130(35.9%)	62(62%)/38(38%)	1.094	0.692-1.728	0.725
	Dominant	GG vs. AA+AG	28(15.5%)/153(84.5%)	6(12%)/44(88%)	1.342	0.523-3.447	0.655
	Recessive	AG+GG vs. AA	102(56.4%)/79(43.6%)	32(64%)/18(36%)	0.726	0.380-1.388	0.419
	Overdominant	AA+GG vs.AG	107(59.1%)/74(40.9%)	24(48%)/26(52%)	1.566	0.835-2.938	0.197
rs6922	Codominant	GG vs.GTvs.TT	65(35.9%)/88(48.6%)/28(15.5%)	23(46%)/21(42%)/6(12%)	-	-	0.431
	Allele contrast	G vs. T	218(60.2%)/144(39.8%)	67(67%)/33(33%)	0.746	0.467-1.189	0.246
	Dominant	TT vs. GG+GT	28(15.5%)/153(84.5%)	6(12%)/44(88%)	1.342	0.523-3.447	0.655
	Recessive	GT+TT vs. GG	116(64.1%)/65(35.9%)	27(54%)/23(46%)	1.520	0.807-2.865	0.249
	Overdominant	GG+TT vs. GT	93(51.4%)/88(48.6%)	29(58%)/21(42%)	0.765	0.406-1.441	0.428
rs4373337	Codominant	AA vs.GA vs. GG	79(43.6%)/74(40.9%)/28(15.5%)	19(38%)/26(52%)/5(10%)	-	-	0.336
	Allele contrast	A vs. G	232(64.1%)/130(35.9%)	64(64%)/36(36%)	1.004	0.633-1.592	1
	Dominant	GG vs. GA+AA	28 (15.5%)/153(84.5%)	5(10%)/45(90%)	1.647	0.601-4.513	0.373
	Recessive	GA+GG vs. AA	102(56.4%)/79(43.6%)	31(62%)/19(38%)	0.791	0.416-1.504	0.520
	Overdominant	AA+GG vs. GA	107(59.1%)/74(40.9%)	24(48%)/26(52%)	1.566	0.835-2.938	0.197

Table 2 Genotypic and allelic distribution of $A_{2A}R$ gene between all patients and controls

SNP ID	Genetic model	Genotype/allele	Cases	Controls	OR	95%CI	*p value*
rs2267076	Codominant	CC vs. TC vs. TT	69(38.1%)/86(47.5%)/26(14.4%)	21(42.0%)/18(36.0%)/11(22.0%)	-	-	0.264
	Allele contrast	C vs. T	224(61.9%)/138(38.1%)	60(60%)/40(40%)	1.082	0.688-1.702	0.817
	Dominant	TT vs. TC+CC	26(14.4%)/155(85.6%)	11(22%)/39(78%)	0.595	0.271-1.307	0.275
	Recessive	TC+TT vs. CC	112(61.9%)/69(38.1%)	29(58%)/21(42%)	1.175	0.622-2.222	0.627
	Overdominant	CC+TT vs. TC	95(52.5%)/86(47.5%)	32(64%)/18(36%)	0.621	0.325-1.187	0.153
rs3761422	Codominant	CC vs. CT vs. TT	71(39.2%)/85(47.0%)/25(13.8%)	21(42.0%)/20(40.0%)/9(18.0%)	-	-	0.643
	Allele contrast	C vs. T	227(52.6%)/135(47.4%)	62(62%)/38(38%)	0.681	0.427-1.085	0.129
	Dominant	TT vs. CT+CC	25(13.8%)/156(86.2%)	9(18%)/41(82%)	0.730	0.316-1.684	0.500
	Recessive	CT+TT vs. CC	110(60.8%)/71(39.2%)	29(58%)/21(42%)	1.122	0.594-2.119	0.746
	Overdominant	CC+TT vs. CT	96(53%)/85(47%)	30(60%)/20(40%)	0.753	0.398-1.423	0.425
rs2298383	Codominant	CC vs. CT vs. TT	43(23.8%)/92(50.8%)/46(25.4%)	12(24%)/19(38%)/19(38%)	-	-	0.181
	Allele contrast	C vs. T	178(49.2%)/184(50.8%)	43(43%)/57(57%)	1.282	0.821-2.004	0.309
	Dominant	TT vs. CT+CC	46(25.4%)/135(74.6%)	19(38%)/31(62%)	0.556	0.287-1.078	0.109
	Recessive	CT+TT vs. CC	138(76.2%)/43(23.8%)	38(76%)/12(24%)	1.013	0.487-2.111	1
	Overdominant	CC+TT vs. CT	89(49.2%)/92(50.8%)	31(62%)/19(38%)	0.593	0.312-1.126	0.113
rs4822492	Codominant	CC vs. CG vs. GG	42(23.2%)/94(51.9%)/45(24.9%)	12(24%)/19(38%)/19(38%)	--		0.144

	Allele contrast	C vs. G	178(49.2%)/184(50.8%)	43(43%)/57(57%)	1.282	0.821-2.004	0.309
	Dominant	GG vs. CG+CC	45(24.9%)/136(75.1%)	19(38%)/31(62%)	0.540	0.278-1.048	0.075
	Recessive	CG+GG vs. CC	139(76.8%)/42(23.2%)	38(76%)/12(24%)	1.045	0.501-2.180	1
	Overdominant	CC+GG vs. CG	87(48.1%)/94(51.9%)	31(62%)/19(38%)	0.567	0.299-1.007	0.109
rs2236624	Codominant	CC vs. CT vs. TT	87(48.1%)/74(40.9%)/20(11.0%)	23(46%)/18(36%)/9(18%)	-	-	0.425
	Allele contrast	C vs. T	248(68.5%)/114(31.5%)	64(64%)/36(36%)	1.224	0.769-1.947	0.401
	Dominant	TT vs. CT+CC	20(11%)/161(89%)	9(18%)/41(82%)	0.566	0.240-1.335	0.227
	Recessive	CT+TT vs. CC	94(51.9%)/87(48.1%)	27(54%)/23(46%)	0.920	0.491-1.725	0.873
	Overdominant	CC+TT vs. CT	107(59.1%)/74(40.9%)	32(64%)/18(36%)	0.813	0.425-1.557	0.625
rs4822489	Codominant	GG vs. GT vs. TT	45(24.9%)/94(51.9%)/42(23.2%)	19(38%)/19(38%)/12(24%)	-	-	0.144
	Allele contrast	G vs. T	184(50.8%)/178(49.2%)	57(51.8%)/43(48.2%)	0.961	0.627-1.473	0.913
	Dominant	TT vs. GT+GG	42(23.2%)/139(76.8%)	12(24%)/38(76%)	0.957	0.459-1.996	1
	Recessive	GT+TT vs. GG	136(75.1%)/45(24.9%)	31(62%)/19(38%)	1.852	0.954-3.595	0.075
	Overdominant	GG+TT vs. GT	87(48.1%)/94(51.9%)	31(62%)/19(38%)	0.567	0.299-1.077	0.109

Table 3 Genotypic and allelic distribution of CD73 gene between all patients and controls in different genders

SNP ID	Gender	Genetic model	Genotype/allele	Cases	Controls	OR	95%CI	*p* value
rs2065114	Male	Codominant	AA vs. GA vs. GG	15(16.3%)/47(51.1%)/30(32.6%)	3(13.6%)/10(45.5%)/9(40.9%)	-	-	0.808
		Allele contrast	A vs. G	77(41.8%)/107(58.2%)	16(36.4%)/28(63.6%)	1.259	0.638-2.487	0.609
		Dominant	GG vs. GA+AA	30(32.6%)/62(67.4%)	9(40.9%)/13(59.1%)	0.699	0.269-1.817	0.618
		Recessive	GA+GG vs. AA	77(83.7%)/15(16.3%)	19(86.4%)/3(13.6%)	0.811	0.213-3.087	1
		Overdominant	AA+GG vs. GA	45(48.9%)/47(51.1%)	12(54.5%)/10(45.5%)	0.798	0.314-2.029	0.813
	Female	Codominant	AA vs. GA vs. GG	14(15.7%)/42(47.2%)/33(37.1%)	4(14.3%)/14(50%)/10(35.7%)	-	-	1
		Allele contrast	A vs. G	70(39.3%)/108(60.7%)	22(39.3%)/34(60.7%)	1.002	0.542-1.852	1
		Dominant	GG vs. GA+AA	33(37.1%)/56(62.9%)	10(35.7%)/18(64.3%)	1.061	0.438-2.569	1
		Recessive	GA+GG vs. AA	75(84.3%)/14(15.7%)	24(85.7%)/4(14.3%)	0.893	0.268-2.972	1
		Overdominant	AA+GG vs. GA	47(52.8%)/42(47.2%)	14(50%)/14(50%)	1.119	0.478-2.617	0.831
rs2229523	Male	Codominant	AAvs.AG vs.GG	14(15.2%)/47(51.1%)/31(33.7%)	3(13.6%)/9(40.9%)10(45.5%)	-	-	0.619
		Allele contrast	A vs. G	75(40.8%)/109(59.2%)	15(34.1%)/29(65.9%)	1.330	0.668-2.650	0.493
		Dominant	GG vs. AG+AA	31(33.7%)/61(66.3%)	10(45.5%)/12(54.5%)	0.610	0.237-1.567	0.330
		Recessive	AG+GG vs. AA	78(84.8%)/14(15.2%)	19(86.4%)/3(13.6%)	0.880	0.229-3.373	1
		Overdominant	AA+GG vs. AG	45(48.9%)/47(51.1%)	13(59.1%)/9(40.9%)	0.663	0.258-1.702	0.479
	Female	Codominant	AAvs.AG vs.GG	14(15.7%)/41(46.1%)/34(38.2%)	3(10.7%)/12(42.9%)/13(46.4%)	-	-	0.732
		Allele contrast	A vs. G	69(38.8%)/109(61.2%)	18(32.1%)/38(67.9%)	1.336	0.707-2.526	0.429
		Dominant	GG vs. AG+AA	34(38.2%)/55 (61.8%)	13(46.4%)/15(53.6%)	0.713	0.303-1.681	0.509

		Recessive	AG+GG vs. AA	75(84.3%)/14(15.7%)	25(89.3%)/3(10.7%)	0.643	0.171-2.422	0.564
		Overdominant	AA+GG vs. AG	48(53.9%)/41(46.1%)	16(57.1%)/12(42.9%)	0.878	0.373-2.068	0.830
rs4579322	Male	Codominant	AAvs.AT vs.TT	30(32.6%)/47(51.1%)/15(16.3%)	9(40.9%)/10(45.5%)/3(13.6%)	-	-	0.808
		Allele contrast	A vs. T	107(58.2%)/77(41.8%)	28(63.6%)/16(36.4%)	0.794	0.402-1.568	0.609
		Dominant	TT vs. AA+AT	15(16.3%)/77(83.7%)	3(13.6%)/19(86.4%)	1.234	0.324-4.700	1
		Recessive	AT+TT vs. AA	62(67.4%)/30(32.6%)	13(59.1%)/9(40.9%)	1.431	0.550-3.719	0.618
		Overdominant	AA+TT vs.AT	45(48.9%)/47(51.1%)	12(54.5%)/10(45.5%)	0.798	0.314-2.029	0.813
	Female	Codominant	AAvs.AT vs.TT	33(37.1%)/42(47.2%)/14(15.7)	10(35.7%)/14(50%)/4(14.3%)	-	-	1
		Allele contrast	A vs. T	108(60.7%)/70(39.3%)	34(60.7%)/22(39.3%)	0.998	0.540-1.846	1
		Dominant	TT vs. AA+AT	14(15.7%)/75(84.3%)	4(14.3%)/24(85.7%)	1.120	0.336-3.728	1
		Recessive	AT+TT vs. AA	56(62.9%)/33(37.1%)	18(17.7%)/10(35.7%)	0.943	0.389-2.283	1
		Overdominant	AA+TT vs.AT	47(52.8%)/42(47.2%)	14(50%)/14(50%)	1.119	0.478-2.617	0.831
rs9444348	Male	Codominant	AA vs. AG vs. GG	11(12%)/38(41.3%)/43(46.7%)	4(18.2%)/9(40.9%)/9(40.9%)	-	-	0.756
		Allele contrast	A vs. G	60(32.6%)/124(67.4%)	17(38.6%)/27(61.4%)	0.769	0.389-1.518	0.480
		Dominant	GG vs. AA+AG	43(46.7%)/49(53.3%)	9(40.9%)/13(59.1%)	1.268	0.493-3.256	0.643
		Recessive	AG+GG vs. AA	81(88%)/11(12%)	18(81.8%)/4(18.2%)	1.636	0.467-5.730	0.484
		Overdominant	AA+GG vs.AG	54(58.7%)/38(41.3%)	13(59.1%)/9(40.9%)	0.984	0.382-2.533	1
	Female	Codominant	AA vs.AGvs.GG	12(13.5%)/32(36%)/45(50.6%)	1(3.6%)/14(50%)/13(46.4%)	-	-	0.245
		Allele contrast	A vs. G	56(31.5%)/122(68.5%)	16(28.6%)/40(71.4%)	1.148	0.593-2.221	0.742
		Dominant	GG vs. AA+AG	45(50.6%)/44(49.4%)	13(46.4%)/15(53.6%)	1.180	0.504-2.764	0.829
		Recessive	AG+GG vs. AA	77(86.5%)/12(13.5%)	27(96.4%)/1(3.6%)	0.238	0.029-1.915	0.186
		Overdominant	AA+GG vs.AG	57(64%)/32(36%)	14(50%)/14(50%)	1.781	0.755-4.201	0.267

rs9450282	Male	Codominant	AA vs.AGvs.GG	39(42.4%)/40(43.5%)/13(14.1%)	7(31.8%)/11(50%)/4(18.2%)	-	-	0.655
		Allele contrast	A vs. G	118(64.1%)/66(35.9%)	25(56.8%)/19(43.2%)	1.359	0.696-2.651	0.389
		Dominant	GG vs. AA+AG	13(14.1%)/79(85.9%)	4(18.2%)/18(81.8%)	0.741	0.216-2.539	0.739
		Recessive	AG+GG vs. AA	53(57.6%)/39(42.4%)	15(68.2%)/7(31.8%)	0.634	0.236-1.703	0.470
		Overdominant	AA+GG vs.AG	52(56.5%)/40(43.5%)	11(50%)/11(50%)	1.300	0.512-3.301	0.638
	Female	Codominant	AA vs.AGvs.GG	40(44.9%)/34(38.2%)/15(16.9%)	11(39.3%)/15(53.6%)/2(7.1%)	-	-	0.269
		Allele contrast	A vs. G	114(64%)/64(36%)	37(66.1%)/19(33.9%)	0.915	0.486-1.721	0.873
		Dominant	GG vs. AA+AG	15(16.9%)/74(83.1%)	2(7.1%)/26(92.9%)	2.635	0.564-12.311	0.355
		Recessive	AG+GG vs. AA	49(55.1%)/40(44.9%)	11(39.3%)/17(60.7%)	1.893	0.796-4.500	0.194
		Overdominant	AA+GG vs.AG	55(61.8%)/34(38.2%)	13(46.4%)/15(53.6%)	1.867	0.792-4.398	0.189
rs6922	Male	Codominant	GG vs.GTvs.TT	31(33.7%)/47(51.1%)/14(15.2%)	10(45.5%)/9(40.9%)/3(13.6%)	-	-	0.555
		Allele contrast	G vs. T	109(59.2%)/75(40.8%)	29(65.9%)/15(34.1%)	0.752	0.377-1.498	0.493
		Dominant	TT vs. GG+GT	14(15.2%)/78(84.8%)	3(13.6%)/19(86.4%)	1.137	0.296-4.359	1
		Recessive	GT+TT vs. GG	61(66.3%)/31(33.7%)	12(54.5%)/10(45.5%)	1.640	0.638-4.215	0.330
		Overdominant	GG+TT vs. GT	45(48.9%)/47(51.1%)	13(59.1%)/9(40.9%)	0.633	0.258-1.702	0.479
	Female	Codominant	GG vs.GTvs.TT	34(38.2%)/41(46.1%)/14(15.7%)	13(46.4%)/12(42.9%)/3(10.7%)	-	-	0.732
		Allele contrast	G vs. T	109(61.2%)/69(38.8%)	38(67.9%)/18(32.1%)	0.748	0.396-1.414	0.429
		Dominant	TT vs. GG+GT	14(15.7%)/75(84.3%)	3(10.7%)/25(89.3%)	1.556	0.413-5.861	0.759
		Recessive	GT+TT vs. GG	55(61.8%)/34(38.2%)	15(53.6%)/13(46.4%)	1.402	0.595-3.303	0.509
		Overdominant	GG+TT vs. GT	48(53.9%)/41(46.1%)	16(57.1%)/12(42.9%)	0.878	0.373-2.068	0.830
rs4373337	Male	Codominant	AA vs.GA vs. GG	39(42.4%)/40(43.5%)/13(14.1%)	9(40.9%)/10(45.5%)/3(13.6%)	-	-	1
		Allele contrast	A vs. G	118(64.1%)/66(35.9%)	28(63.6%)/16(36.4%)	1.022	0.515-2.025	1

	Dominant	GG vs. GA+AA	13(14.1%)/79(85.9%)	3(13.6%)/19(86.4%)	1.042	0.270-4.027	1
	Recessive	GA+GG vs. AA	53(57.6%)/39(42.4%)	13(59.1%)/9(40.9%)	0.941	0.366-2.421	1
	Overdominant	AA+GG vs. GA	52(56.5%)/40(43.5%)	12(54.5%)/10(45.5%)	1.083	0.425-2.759	1
Female	Codominant	AA vs.GA vs. GG	40(44.9%)/34(38.2%)/15(16.9%)	10(35.7%)/16(57.1%)/2(7.1%)	-	-	0.206
	Allele contrast	A vs. G	114(64%)/64(36%)	36(64.3%)/20(35.7%)	0.990	0.529-1.852	1
	Dominant	GG vs. GA+AA	15(16.9%)/74(83.1%)	2(7.1%)/26(92.9%)	2.635	0.564-12.311	0.355
	Recessive	GA+GG vs. AA	49(55.1%)/40(44.9%)	18(64.3%)/10(35.7%)	0.681	0.283-1.639	0.512
	Overdominan	AA+GG vs. GA	55(61.8%)/34(38.2%)	12(42.9%)/16(57.1%)	2.157	0.911-5.107	0.805

Table 4 Genotypic and allelic distribution of $A_{2A}R$ gene between all patients and controls in different genders

SNP ID	Gender	Genetic model	Genotype/allele	Cases	Controls	OR	95%CI	*p value*
rs2267076	Female	Codominant	CC vs. TC vs. TT	32(36.3%)/49(55.7%)/7(8%)	14(50%)/8(28.6%)/6(21.4%)	-	-	0.019*
		Allele contrast	C vs. T	113(64.2%)/63(35.8%)	36(64.3%)/20(35.7%)	0.996	0.532-1.866	1
		Dominant	TT vs. TC+CC	7(8%)/81(92%)	6(21.4%)/22(78.6%)	0.317	0.097-1.039	0.079
		Recessive	TC+TT vs. CC	56(63.6%)/32(36.4%)	14(50%)/14(50%)	1.750	0.741-4.130	0.267
		Overdominant	CC+TT vs. TC	39(44.3%)/49(55.7%)	20(71.4%)/8(28.6%)	0.318	0.127-0.800	0.017*
	Male	Codominant	CC vs. TC vs. TT	37(39.8%)/37(39.8%)/19(20.4%)	7(31.8%)/10(45.5%)/5(22.7%)	-	-	0.823
		Allele contrast	C vs. T	111(59.7%)/75(40.3%)	24(54.5%)/20(45.5%)	1.233	0.636-2.391	0.610
		Dominant	TT vs. TC+CC	19(20.4%)/74(79.6%)	5(22.7%)/17(77.3%)	0.823	0.286-2.668	1
		Recessive	TC+TT vs. CC	56(60.2%)/37(39.8%)	15(68.2%)/7(31.8%)	0.706	0.263-1.898	0.627
		Overdominant	CC+TT vs. TC	56(60.2%)/37(39.8%)	12(54.5%)/10(45.5%)	1.261	0.495-3.217	0.638
rs3761422	Female	Codominant	CC vs. CT vs. TT	34(38.6%)/47(53.4%)/7(8%)	14(50%)/10(35.7%)/4(14.3%)	-	-	0.250
		Allele contrast	C vs. T	121(66.5%)/61(33.5%)	38(67.9%)/18(32.1%)	0.940	0.496-1.781	0.873
		Dominant	TT vs. CT+CC	7(8%)/81(92%)	4(14.3%)/24(85.7%)	0.519	0.140-1.922	0.457
		Recessive	CT+TT vs. CC	54(61.4%)/34(38.6%)	14(50%)/14(50%)	1.588	0.675-3.739	0.379
		Overdominant	CC+TT vs. CT	41(46.6%)/47(53.4%)	18(64.3%)/10(35.7%)	0.485	0.201-1.167	0.130
	Male	Codominant	CC vs. CT vs. TT	37(39.8%)/38(40.9%)/18(19.4%)	7(31.8%)/10(45.5%)/5(22.7%)	-	-	0.823

		Allele contrast	C vs. T	112(60.2%)/74(39.8%)	24(54.5%)/20(45.5%)	1.261	0.651-2.445	0.500
		Dominant	TT vs. CT+CC	18(19.4%)/75(80.6%)	5(22.7%)/17(77.3%)	0.816	0.266-2.506	0.769
		Recessive	CT+TT vs. CC	56(60.2%)/37(39.8%)	15(68.2%)/7(31.8%)	0.706	0.263-1.898	0.627
		Overdominant	CC+TT vs. CT	55(59.1%)/38(40.9%)	12(54.5%)/10(45.5%)	1.206	0.473-3.074	0.811
rs2298383	Female	Codominant	CC vs. CT vs. TT	15(17%)/52(59.1%)/21(23.9%)	7(25%)/9(32.1%)/12(42.9%)	-	-	0.039*
		Allele contrast	C vs. T	82(46.6%)/94(53.4%)	23(41.1%)/33(58.9%)	1.252	0.681-2.302	0.538
		Dominant	TT vs. CT+CC	21(23.9%)/67(76.1%)	12(42.9%)/16(57.1%)	0.418	0.171-1.022	0.059
		Recessive	CT+TT vs. CC	73(83%)/15(17%)	21(75%)/7(25%)	1.622	0.585-4.499	0.408
		Overdominant	CC+TT vs. CT	36(40.9%)/52(59.1%)	19(67.9%)/9(32.1%)	0.328	0.133-0.806	0.017*
	Male	Codominant	CC vs. CT vs. TT	28(30.1%)/40(43%)/25(26.9%)	5(22.7%)/10(45.5%)/7(31.8%)	-	-	0.830
		Allele contrast	C vs. T	96(51.6%)/90(48.4%)	20(45.5%)/24(54.5%)	1.280	0.662-2.475	0.505
		Dominant	TT vs. CT+CC	25(26.9%)/68(73.1%)	7(31.8%)/15(68.2%)	0.788	0.288-2.158	0.792
		Recessive	CT+TT vs. CC	65(69.9%)/28(30.1%)	17(77.3%)/5(22.7%)	0.683	0.229-2.033	0.605
		Overdominant	CC+TT vs. CT	53(57%)/40(43%)	12(54.5%)/10(45.5%)	1.104	0.434-2.810	1
rs4822492	Female	Codominant	CC vs. CG vs. GG	15(17%)/53(60.2%)/20(22.7%)	7(25%)/9(32.1%)/12(42.9%)	-	-	0.029*
		Allele contrast	C vs. G	83(47.2%)/93(52.8%)	23(41.1%)/33(58.9%)	1.281	0.696-2.355	0.445
		Dominant	GG vs. CG+CC	20(22.7%)/68(77.3%)	12(42.9%)/16(57.1%)	0.392	0.160-0.964	0.052
		Recessive	CG+GG vs. CC	73(83%)/15(17%)	21(75%)/7(25%)	1.622	0.585-4.499	0.408

		Overdominant	CC+GG vs. CG	35(39.8%)/53(60.2%)	19(67.9%)/9(32.1%)	0.313	0.127-0.770	0.016*
	Male	Codominant	CC vs. CG vs. GG	27(29%)/41(44.1%)/25(26.9%)	5(22.7%)/10(45.5%)/7(31.8%)	-	-	0.871
		Allele contrast	C vs. G	95(51.1%)/91(48.9%)	20(45.5%)/24(54.5%)	1.253	0.648-2.422	0.615
		Dominant	GG vs. CG+CC	25(26.9%)/68(73.1%)	7(31.8%)/15(68.2%)	0.788	0.288-2.158	0.792
		Recessive	CG+GG vs. CC	66(71%)/27(29%)	17(77.3%)/5(22.7%)	0.719	0.241-2.145	0.610
		Overdominant	CC+GG vs. CG	52(55.9%)/41(44.1%)	12(54.5%)/10(45.5%)	1.057	0.415-2.689	1
rs2236624	Female	Codominant	CC vs. CT vs. TT	44(50%)/39(44.3%)/5(5.7%)	15(53.6%)/8(28.6%)/5(17.8%)	-	-	0.072
		Allele contrast	C vs. T	127(72.2%)/49(27.8%)	38(67.9%)/18(32.1%)	1.228	0.641-2.353	0.612
		Dominant	TT vs. CT+CC	5(5.7%)/83(94.3%)	5(17.9%)/23(82.1%)	0.277	0.074-1.040	0.060
		Recessive	CT+TT vs. CC	44(50%)/44(50%)	13(46.4%)/15(53.6%)	1.154	0.492-2.705	0.829
		Overdominant	CC+TT vs. CT	49(55.7%)/39(44.3%)	20(71.4%)/8(28.6%)	0.503	0.200-1.263	0.186
	Male	Codominant	CC vs. CT vs. TT	43(46.2%)/35(37.7%)/15(16.1%)	8(36.4%)/10(45.5%)/4(18.2%)	-	-	0.734
		Allele contrast	C vs. T	121(65.1%)/65(34.9%)	26(59.1%)/18(40.9%)	1.289	0.658-2.524	0.488
		Dominant	TT vs. CT+CC	15(16.1%)/78(83.9%)	4(18.2%)/18(81.8%)	0.865	0.256-2.920	1
		Recessive	CT+TT vs. CC	50(53.8%)/43(46.2%)	14(63.6%)/8(36.4%)	0.664	0.255-1.735	0.478
		Overdominant	CC+TT vs. CT	58(62.4%)/35(37.6%)	12(54.5%)/10(45.5%)	1.381	0.540-3.529	0.628
rs4822489	Female	Codominant	GG vs. GT vs. TT	20(22.7%)/53(60.2%)/15(17%)	12(27.6%)/9(53.4%)/7(19%)	-	-	0.029*
		Allele contrast	G vs. T	93(52.8%)/83(47.2%)	33(58.9%)/23(41.1%)	0.781	0.425-1.436	0.445

	Dominant	TT vs. GT+GG	15(17%)/73(83%)	7(25%)/21(75%)	0.616	0.222-1.710	0.408
	Recessive	GT+TT vs. GG	68(77.3%)/20(22.7%)	16(57.1%)/12(42.9%)	2.550	1.037-6.268	0.052
	Overdominant	GG+TT vs. GT	35(39.8%)/53(60.2%)	19(67.9%)/9(32.1%)	0.313	0.127-0.770	0.016*
Male	Codominant	GG vs. GT vs. TT	25(26.9%)/41(44.1%)/27(25.9%)	7(31.8%)/10(45.5%)/5(22.7%)	-	-	0.871
	Allele contrast	G vs. T	91(48.9%)/95(51.1%)	24(54.5%)/20(45.5%)	0.798	0.413-1.543	0.615
	Dominant	TT vs. GT+GG	27(29%)/66(71%)	5(22.7%)/17(77.3%)	1.391	0.466-4.150	0.610
	Recessive	GT+TT vs. GG	68(73.1%)/25(26.9%)	15(68.2%)/7(31.8%)	1.269	0.463-3.476	0.792
	Overdominant	GG+TT vs. GT	52(55.9%)/41(44.1%)	12(54.5%)/10(45.5%)	1.057	0.415-2.689	1

Table 5 Genotypic and allelic distribution of CD73 and $A_{2A}R$ genes between patients with negative intracranial imaging and positive intracranial imaging

Gene	SNP ID	Genetic model	Genotype/allele	negative intracranial imaging	positive intracranial imaging	OR	95%CI	*p value*
CD73	rs4431401	Codominant	CC vs. CT vs.TT	16(15.8%)/36(35.6%)/49(48.5%)	7(8.8%)/38(47.5%)/35(43.8%)	-	-	0.173
		Allele contrast	C vs. T	68(33.7%)/134(66.3%)	52(32.5%)/108(67.5%)	1.504	0.678-1.638	0.823
$A_{2A}R$	rs2267076	Codominant	CC vs. TC vs. TT	36(35.6%)/49(48.5%)/16(15.8%)	33(41.3%)/37(46.3%)/10(12.5%)	-	-	0.705
		Allele contrast	C vs. T	121(59.9%)/81(40.1%)	103(64.4%)/57(35.6%)	0.827	0.538-1.269	0.446
$A_{2A}R$	rs2298383	Codominant	CC vs. CT vs. TT	24(23.8%)/54(53.5%)/23(22.8%)	19(23.8%)/38(47.5%)/23(28.7%)	-	-	0.642
		Allele contrast	C vs. T	102(50.5%)/100(49.5%)	76(47.5%)/84(52.5%)	1.127	0.744-1.708	0.598
$A_{2A}R$	rs4822492	Codominant	CC vs. CG vs. GG	23(23.8%)/56(53.5%)/22(22.8%)	19(23.8%)/38(47.5%)/23(28.7%)	-	-	0.499
		Allele contrast	C vs. G	102(50.5%)/100(49.5%)	76(47.5%)/84(52.5%)	1.127	0.744-1.708	0.598
$A_{2A}R$	rs4822489	Codominant	GG vs.GT vs. TT	22(21.8%)/56(55.4%)/23(22.8%)	23(28.7%)/38(47.5%)/19(23.8%)	-	-	0.499
		Allele contrast	G vs. T	100(49.5%)/102(50.5%)	84(52.5%)/76(47.5%)	0.887	0.586-1.343	0.598

Table 6 Genotypic and allelic distribution of CD73 and $A_{2A}R$ genes between patients with epileptic seizure frequencies < 2 times/ year and patients with epileptic seizure frequencies ⩾2 times/ year

Gene	SNP ID	Genetic model	Genotype/allele	Epileptic seizure frequencies < 2 times/ year	Epileptic seizure frequencies ⩾2 times/ year	OR	95%CI	*p value*
CD73	rs4431401	Codominant	CC vs. CT vs.TT	9(11.3%)/35(43.8%)/36(45%)	14(13.9%)/39(38.6%)/48(47.5%)	-	-	0.761
		Allele contrast	C vs. T	53(33.1%)/107(66.9%)	67(33.2%)/135(66.8%)	0.998	0.642-1.551	1
A2AR	rs2267076	Codominant	CC vs. TC vs. TT	28(35%)/36(45%)/16(20%)	41(40.6%)/50(49.5%)/10(9.9%)	-	-	0.165
		Allele contrast	C vs. T	92(57.5%)/68(42.5%)	132(65.3%)/70(34.7%)	0.717	0.468-1.099	0.129
A2AR	rs2298383	Codominant	CC vs. CT vs. TT	22(27.5%)/38(47.5%)/20(25%)	21(20.8%)/54(53.5%)/26(25.7%)	-	-	0.570
		Allele contrast	C vs. T	82(51.2%)/78(48.8%)	96(47.5%)/106(52.5%)	1.161	0.766-1.758	0.526
A2AR	rs4822492	Codominant	CC vs. CG vs. GG	22(27.5%)/39(48.8%)/19(23.8%)	20(19.8%)/55(54.5%)/26(25.7%)	-	-	0.489
		Allele contrast	C vs. G	83(51.9%)/77(48.1%)	95(47%)/107(53%)	1.214	0.801-1.839	0.397
A2AR	rs4822489	Codominant	GG vs.GT vs. TT	19(23.8%)/39(48.8%)/22(27.5%)	26(25.7%)/55(54.5%)/20(19.8%)	-	-	0.489
		Allele contrast	G vs. T	77(48.1%)/83(51.9%)	107(53%)/95(47%)	0.824	0.544-1.248	0.397
